# Aprepitant plus palonosetron for the prevention of postoperative nausea and vomiting after breast cancer surgery: a double blind, randomized trial

**DOI:** 10.6061/clinics/2020/e1688

**Published:** 2020-08-28

**Authors:** Thiago Ramos Grigio, Angela Maria Sousa, Gabriel Guimarães Nunes Magalhães, Hazem Adel Ashmawi, Joaquim Edson Vieira

**Affiliations:** I Anestesia, Instituto do Cancer do Estado de Sao Paulo (ICESP), Hospital das Clinicas HCFMUSP, Faculdade de Medicina, Universidade de Sao Paulo, Sao Paulo, SP, BR.; II Anestesia, Hospital das Clinicas HCFMUSP, Faculdade de Medicina, Universidade de Sao Paulo, Sao Paulo, SP, BR.; III Campus Darcy Ribeiro, Faculdade Medicina, Universidade de Brasilia, Brasilia, DF, BR.; IV Anestesiologia Experimental LIM-08, Hospital das Clinicas HCFMUSP, Faculdade de Medicina, Universidade de Sao Paulo, Sao Paulo, SP, BR.; V Cirurgia, Faculdade de Medicina FMUSP, Universidade de Sao Paulo, Sao Paulo, SP, BR.

**Keywords:** Postoperative Nausea and Vomiting, Mastectomy, Segmental Aprepitant Palonos

## Abstract

**OBJECTIVES:**

To evaluate the addition of a fourth antiemetic intervention in patients at high risk for postoperative nausea and vomiting (PONV).

**METHODS:**

High-risk patients (Apfel score 3 or 4) scheduled for unilateral mastectomy were randomly allocated in one of two groups, oral aprepitant (oral aprepitant 80 mg, intravenous dexamethasone 8 mg, and palonosetron 0.075 mg) and oral placebo (oral placebo, intravenous dexamethasone 4 mg, and palonosetron 0.075 mg). Patients and caregivers were blinded to the group assignments. The primary efficacy endpoints included the incidence of nausea and vomiting, and the secondary endpoints included use of rescue antiemetics during a 48-hour postoperative period. ClinicalTrials.gov: NCT02431286.

**RESULTS:**

One hundred patients were enrolled in this study and 91 were analyzed, 48 in group A and 43 in group P. No patient presented with nausea or vomiting in the first 2 hours after surgery. From the 2^nd^ to the 6^th^ hour, the incidence of PONV was 8.33% in group A and 9.30% in group P. In the first 24 hours, the incidence of PONV was 27.08% in the group A and 20.93% in group P. From the 24^th^ to the 48^th^ hour, the incidence of PONV was 8.33% in group A and 13.95% in group P. There were no statistically significant differences in PONV between groups.

**CONCLUSION:**

The addition of aprepitant as a third antiemetic resulted in no significant reduction in the incidence of PONV in this population. However, the incidence of PONV was reduced in relation to the general population.

## INTRODUCTION

Surgery is the primary treatment for women with early-stage breast cancer. Although medically necessary, both excisional breast biopsy and breast-conserving surgery can be emotionally devastating for women. Most of these patients are non-smokers who use opioids in the postoperative period (1), which are known risk factors for postoperative nausea and vomiting (PONV). The four risk factors included in the Apfel Risk Score are female sex, prior history of motion sickness, or PONV, nonsmoking, and the use of postoperative opioids (2). In addition, elevated levels of distress may increase the severity of PONV (3,4) in the postoperative period. Concerns about changes in appearance and scarring, anesthesia, surgical procedures, diagnosis, and prognosis determine such distress. Besides distress, the frequency of PONV is higher among women (20% more nausea and vomiting than male patients) (5,6).

When it comes to breast surgery, a recent work reported 29% of nausea in the post-anesthesia care unit and 35% of post-discharge nausea and vomiting (PDNV) (7) in patients who received prophylactic antiemetic. In a previous study, the incidence of PONV in patients who underwent mastectomy was 38.1%, even with double antiemetic therapy (8). When no prophylaxis was used, the overall incidence of nausea and vomiting during the first 24 hours after surgery was reported to be 75% (9).

This study aimed to determine whether combination therapy with extended-duration antiemetics, palonosetron, and aprepitant is more effective than palonosetron plus dexamethasone in preventing PONV after breast cancer surgery.

## MATERIALS AND METHODS

This protocol was elaborated according to CONSORT statements, 2010 (10). This is a double-blind, placebo-controlled, parallel-group study conducted in the Cancer Institute of the State of São Paulo, Brazil after approval from the Hospital Ethics Committee. The study was registered in Clinical Trials.gov on 01/31/2015 (registration number: NCT02431286)

The drugs used in the study (aprepitant and placebo) were prepared in identical capsules stored in packages labeled A or B by the Central Pharmacy of Hospital das Clinicas of the University of Sao Paulo School of Medicine. The placebo was matched to the study drug for taste, color, and size, and contained starch.

After randomizing the 100 patients according to a random number generator (www.random.org), the description of each group containing the details of the intervention (if capsule A or B) was stored in a sealed opaque envelope.

In the preoperative visit, adult female patients aged between 18 and 55 years scheduled for breast cancer surgery were invited to participate in this study. They were classified as high-risk for PONV according to their Apfel score, and included in the study after written informed consent was obtained. They were randomly distributed into one of two groups (A or B) according to previous sequential randomization. On the day of the surgery, a nurse, not involved in the study, opened the sequential envelope and administered one of the capsules to the patient being studied. The anesthetist in charge of the case, the investigator, and the patient were not informed of which group the patient was in. At the end of the data collection, the groups A and B were identified, and we considered the group that received placebo to be the control group.

All patients fasted starting at midnight, and an intravenous line was inserted the night before the surgery. After entering the operating room, the standard monitoring methods, which included electrocardiography (ECG), non-invasive blood pressure (NIBP), pulse oximetry, capnography, and bispectral index were applied. Patients in both groups received total intravenous anesthesia. First, 2 mg of midazolam was intravenously injected as the pre-anesthetic medication. Thereafter, 2 mg/kg propofol was intravenously injected, followed by 3-5 mcg/kg fentanyl, 4 mg of dexamethasone, and 0.075 mg of palonosetron. Finally, after confirmation of the patient’s loss of consciousness, 0.15 mg/kg cisatracurium was intravenously injected to facilitate tracheal intubation. Mechanical ventilation maintained the end-tidal carbon dioxide partial pressure (ETCO_2_) between 35 and 40 mmHg. Anesthesia was maintained with 1-2 mcg/kg/h remifentanil and propofol 2-3 mg/kg/h adjusted to maintain NIBP and heart rate (HR), approximately 30% lower than the preoperative values. The depth of anesthesia was evaluated by pupillary diameter in response to light, recorded every 10 minutes. Both drugs were infused using Orchestra Module DPS (Fresenius, France). If it were necessary to reverse muscle relaxation, 15 mcg/kg atropine and 0.04-0.07-mg/kg neostigmine were injected.

After the end of the surgery, the patients were transported to the post-anesthesia care unit (PACU), where they stayed until they met the minimum criteria for discharge (11). An intravenous patient-controlled analgesia (PCA) device with a morphine solution (1 mg/ml) was installed after the patient arrived in the PACU. During this stay, patients were monitored according to the institutional protocol (pain according to the visual analog scale from 0 to 10, where 0=no pain and 10=worst possible pain, continuous SpO_2_, NIBP, HR, nausea, and vomiting). Patients were instructed to ask for antiemetic medication every time they felt nauseated. If any nausea, emetic episodes, or both occurred in the first 48 hours postoperatively, 0.625 mg of droperidol *prn* was prescribed as a rescue medication.

After the 2^nd^, 6^th^, 24^th^, and 48^th^ postoperative hours, trained investigators recorded the number of emetic episodes during the preceding interval, and patients orally rated their worst nausea episode on an 11-point numerical scale, where 0 represented no nausea and 10 represented the worst possible nausea. Severe nausea was defined as numerical rating scale (NRS) of 7 or higher, and severe emesis was defined as three or more episodes of vomiting in the period.

We recorded the total dose of rescue antiemetic (droperidol) administered, the intensity of pain and the consumption of morphine in the first 24 hours, the duration of anesthesia and surgery and any side effects reported by the patient.

The primary outcome was the incidence of any nausea, emetic episodes (retching or vomiting), or both (*i.e.*, PONV) during the first 24 postoperative hours. The secondary outcome was the incidence of PONV during the first 48 postoperative hours.

**Theory**: Adding aprepitant to palonosetron and dexamethasone should be more effective than dexamethasone/palonosetron alone.

### Statistical Analysis

We expected a 45% incidence of PONV in the control group (double therapy), a delta up of 30%, and no inferiority margin. We expected a type I error α=0.05/3 (three main outcomes nausea, vomiting and PONV), error II (β=0.2, power 80%), 95% confidence interval and two-tailed hypothesis test. FunctionTwoSamplesProportion.NIS from package TrialSize for R was used with those parameters, estimating 45 patients each group. We planned 10 additional subjects considering possible exclusions.

The results were analyzed with R version 3.4.3 (v18.0) and are expressed as mean±SD. The statistical analysis was performed using the chi-square test and Fisher’s exact test to compare the categorical variables. The Wilcoxon-Mann-Whitney test was used to analyze the number of emetic episodes during the first 24 postoperative hours.

The null hypothesis was ruled out if *p*<0.05. Statistical significance was set at *p*<0.05 for all tests.

## RESULTS

One hundred patients were enrolled in this study, from July 2014 to May 2017, and 50 patients were allocated in each group. Patients in Group A received aprepitant (Aprepitant Group) and Group B received placebo (Placebo Group).

In the placebo group, 50 patients received the study medication. Five patients received ondansetron instead of palonosetron in the operating room (protocol violation). After surgery, one patient was discharged before 24 hours, and was lost to follow-up, and one patient was discharged from the study because he received ondansetron every 8 hours (protocol violation).

**Figure 1:**
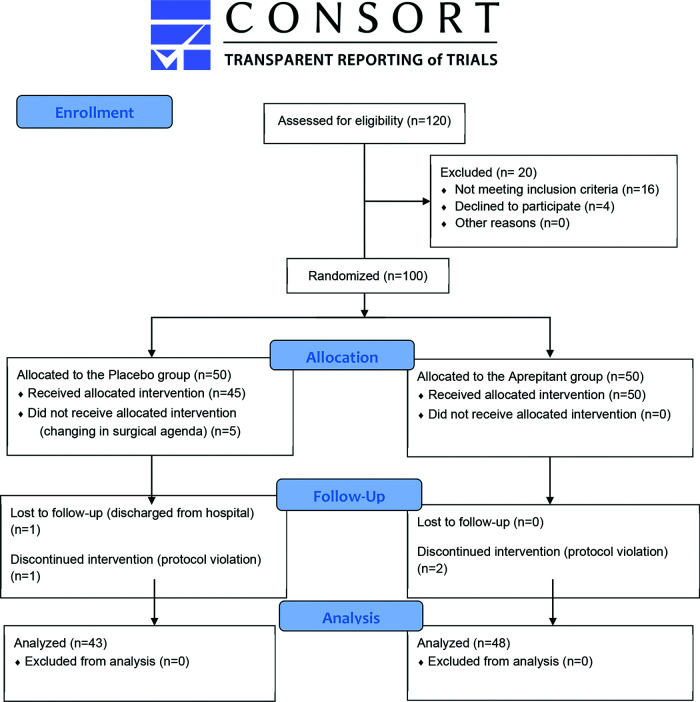
CONSORT 2010 Flow Diagram.

In the aprepitant group, 50 patients received the study medication. In the postoperative period, 3 patients received ondansetron in an 8-hour regimen (protocol violation). Therefore, 91 patients were analyzed: 43 in the placebo group and 48 in the aprepitant group (Figure 1).

There were no statistically significant differences in age, weight, height, Apfel score, or duration of surgery or anesthesia between groups (Table 1). There were no differences in the dose of opioids used in the operating room or the consumption of morphine administered via the PCA device during the entire period of observation (Table 2).

No patient presented with nausea or vomiting between the end of the surgery and 2 postoperative hours (0-2 hours) (Table 3).

From the 2^nd^ to the 6^th^ hour, four patients in the placebo group (9.3%) had nausea, and two vomited (one episode each) (4.65%). In the aprepitant group, four patients (8.33%) had nausea, and one patient (2.08%) vomited (Table 3).

Within the first 24 hours, nine patients in the placebo group (20.93%) had nausea and six patients (13.95%) vomited. In the aprepitant group, 13 patients had nausea (27.08%) and 5 patients (10.41%) vomited (Table 3).

From 24^th^ to the 48^th^ hour, six patients (13.9%) in the placebo group had nausea and two patients (4.65%) vomited. In the aprepitant group, four patients (8.33%) had nausea, and three patients (6.25%) vomited. One patient belonging to aprepitant group had severe emesis (4 episodes of vomiting) from the 6^th^ to the 24^th^ hour and (3 episodes of vomiting) from the 24^th^ to the 48^th^ hour (Table 4).

Two patients in the placebo group and one patient in the aprepitant group needed rescue antiemetic medication during the first 24 hours (0-24 hours).

There were no statistically significant differences between the groups during any period.

## DISCUSSION

Our results showed that the addition of oral aprepitant to palonosetron plus dexamethasone did not reduce postoperative nausea and vomiting in female patients undergoing mastectomy and already receiving two antiemetics. However, the incidence of PONV in this study population was lower than the incidence of PONV previously reported in our institution with patients undergoing mastectomy and receiving the institutional protocol (8).

Palonosetron has a higher binding affinity to serotonin receptors (5HT3) and a longer plasma half-life than other 5-HT3 receptor antagonists. Different researchers reported evidence of receptor internalization (12) and crosstalk between neurokinin-1 (NK1) and 5-HT3 receptor-signaling pathways (13). This raises the possibility that palonosetron’s efficacy in treating delayed emesis could be due to differential inhibition of the 5-HT3/NK1 receptor crosstalk (13).

The idea that palonosetron can enhance NK1 receptor antagonist effects, while ondansetron and granisetron cannot, has been supported by a recent study (14). The use of palonosetron with an NK1 receptor antagonist (aprepitant) during the administration of highly emetogenic chemotherapy reduced the risk of uncontrolled chemotherapy-induced nausea and vomiting (CINV) when compared to other 5-HT3 receptor antagonists (ondansetron, granisetron, and dolasetron) plus aprepitant (14). Our results did not confirm the benefit of such an association. In contrast, in a previous study we reported a reduced incidence of PONV with the combination of ondansetron and aprepitant in patients undergoing laparoscopic surgery (15). Although we did not find a beneficial effect on the association of palonosetron and aprepitant in the present work, the incidence of PONV was lower than that reported in other studies (16), with a very low incidence of vomiting, similar to Yoo et al. (17).

The reason for not identifying a positive effect in the current population could be the number of patients studied, or the population itself. With the current incidence of PONV (20.93% in control, *versus* 27.08% in the aprepitant group), we should include 127 patients from each group to see any significant difference. In fact, we underestimated the effect of palonosetron in reducing PONV in the control group. Another bias that may have occurred in this study is the population itself. Although all patients were considered to be at high risk for PONV (Apfel Score 3 or 4), the use of at least three antiemetic strategies in the two groups prevented the evidence of any difference between treatments. In addition, we did not select patients who had undergone previous chemotherapy-induced nausea and vomiting, a known risk factor for PONV (8). We cannot rule out the hypothesis that the selection of patients with a previous history of CINV had a different outcome. In fact, a previous study reported that palonosetron was more effective than ondansetron in the prevention of PONV in post-chemotherapy ovarian cancer surgeries (18). History of CINV increases the chance of PONV and reclassifies patients regarding the risk of nausea and vomiting. Thus, the difference between antiemetic treatments would be more evident.

However, we could hypothesize that palonosetron is able to reduce the baseline risk of PONV in a significant way, preventing the drug association effect of aprepitant and palonosetron due to its unique pharmacological profile. As shown before, palonosetron has a different mechanism of action than ondansetron, modulating NK1 receptors and decreasing the need to add a specific NK1 receptor antagonist, such as aprepitant (13).

## CONCLUSION

In conclusion, a combination of aprepitant and palonosetron did not reduce the incidence of PONV to a greater extent than palonosetron alone in the first 24 hours after surgery in female patients undergoing mastectomy. We now know that patients submitted to previous chemotherapy are of major risk of PONV, and we should take this into consideration in future studies, including only patients submitted to previous chemotherapy. However, the incidence of PONV was reduced in both groups, compared to the overall incidence of PONV in our institution.

## AUTHOR CONTRIBUTIONS

Grigio TR developed the theory and performed the computations. Sousa AM and Magalhães GGN conceived the study. Vieira JE verified the analytical methods. Ashmawi HA supervised the findings of this work. All of the authors discussed the results and contributed to the final version of the manuscript

## Figures and Tables

**Table 1:** Baseline Characteristics.

Characteristics	Control group (n=43)	Aprepitant group (n=48)
Age	52 (30-81)	53 (26-82)
Weight	66 (41-111)	67 (42-136)
Height	157 (139-175)	159 (145-168)
Apfel score	3 (3-4)	3 (3-4)
Duration of surgery	194.12 (60-585)	301.04 (100-810)
Duration of anesthesia	245.97 (115-640)	235.2 (70-760)

Data are presented as the mean OR and median (interval). Placebo group: oral placebo plus IV dexamethasone and palonosetron. Aprepitant group: oral aprepitant plus IV dexamethasone and palonosetron. There were no significant differences between groups.

**Table 2:** Opioid Consumption during surgery or in the postoperative period.

	Placebo			Aprepitant			*p*
	n	Mean±SD	Median (interval)	n	Mean±SD	Median (interval)	
Fentanyl (mcg)	24	364.6±179.1	300 (150-950)	18	444.4±200.7	350 (250-900)	0.12
Morphine (mg)	13	4.9±2.1	4 (3-10)	10	4.1±2	4 (2-8)	0.35
Morphine consumption (postoperative period) (mg): 0-24 h	34	3.84±4.34	2.5 (0-16)	39	5.35±6.8	3 (0-26)	0.16

Data are presented as the mean±SD. Placebo group: oral placebo plus IV dexamethasone and palonosetron. Aprepitant group: oral aprepitant plus IV dexamethasone and palonosetron. Student’s t test was used to analyze the postsurgical morphine consumption. All other analyses were performed with the Mann-Whitney test. OR=operating room; PCA=patient-controlled analgesia; SD=standard deviation.

**Table 3:** Incidence of Nausea and Vomiting during the First 48 hours after Surgery.

Time after surgery	Placebo group (n=43)	Aprepitant group (n=48)	*p*-value
0-2 h			
Nausea	0	0	1
Vomiting	0	0	
2-6 h			
Nausea	4 (9.3%)	4 (8.33%)	1
Vomiting	2 (4.65%)	1 (2.08%)	1
PONV	4 (4.65%)	1 (2.08%)	
0-24 h			
Nausea	9 (20.93%)	13 (27.08%)	0.62
Vomiting	6 (13.95%)	5 (10.41%)	0.836
PONV	6 (13.95%)	5 (10.41%)	
24-48 h			
Nausea	6 (13.95%)	4 (8.3%)	0.50
Vomiting	2 (4.65%)	3 (6.25%)	0.66
PONV	2 (4.65%)	3 (6.25%)	

Data are presented as the number of patients (%). Placebo group: oral placebo *plus* IV dexamethasone and palonosetron. Aprepitant group: oral aprepitant *plus* IV dexamethasone and palonosetron. Fisher’s exact test was used for the categorical variables. PONV: postoperative nausea and vomiting.

**Table 4:** Number of Emetic Episodes during the First 24 hours after Surgery.

	Placebo	Aprepitant	*p*
Number of emetic episodes 2-6 h			0.47
None	N=42 (94.1%)	N=47 (97.4%)	
One	N=1 (5.88%)	N=1 (2.56%)	
Number of emetic episodes 0-24 h			0.55
None	N=35 (91.17%)	N=43 (89.74%)	
One	N=3 (5.88%)	N=3 (7.69%)	
Two	N=2 (2.94%)	N=1	
Four (Severe vomiting)	N=0	N=1 (2.56%)	

Data are presented as the number of patients (%). Placebo group: oral placebo plus IV dexamethasone and palonosetron. Aprepitant group: oral aprepitant plus IV dexamethasone and palonosetron. The chi-squared test was used to analyze the severity of nausea; chi-squared and Fisher’s exact tests were used to compare the groups. *p*>0.05 between groups.
